# *Typhula* cf. *subvariabilis*, new snow mould in Antarctica

**DOI:** 10.1080/21501203.2017.1343753

**Published:** 2017-06-23

**Authors:** Yuka Yajima, Motoaki Tojo, Bo Chen, Tamotsu Hoshino

**Affiliations:** a College of Environmental Technology, Muroran Institute of Technology, Muroran, Japan; b Graduate School of Life and Environmental Sciences, Osaka Prefecture University, Sakai, Japan; c SOA Key Laboratory for Polar Science, Polar Research Institute of China, Shanghai, China; d Research Institute of Sustainable Chemistry, National Institute of Advanced Industrial Technology (AIST), Higashi-Hiroshima, Japan; e Graduate School of Advanced Sciences of Matter, Hiroshima University, Higashi-Hiroshima, Japan; f Graduate School of Life and Environmental Sciences, University of Tsukuba, Tsukuba, Japan

**Keywords:** Cryophilic fungi, environmental adaptation, evolution, habitat, psychrophile

## Abstract

We collected snow blight of moss, *Polytrichum juniperinum* on King George Island, maritime Antarctica. Host died in a circle of about 10–30 cm after snow melts. Clamp connected hyphae and no sclerotia were observed on tip of host leaves. DNA sequence of ITS region from moss symptoms were perfectly matched with fruit bodies of *Typhula* sp. on Macquarie Island in the maritime Antarctica and high homology with *Typhula* cf. *subvariabilis* from Iran. Therefore, we suggested that *T*. cf. *subvariabilis* caused snow blight on moss in Antarctica, and this is first record of *Typhula* snow blight in Southern Hemisphere. These results also suggested that fungi in same genera gained similar ecological niche in both Polar Regions.

## Introduction

Snow moulds are cryophilic (cold-adapted) fungi (Hoshino and Matsumoto 2012, 2013) and fungal-like microbes, and they attack dormant plants such as forage crops, winter cereals and conifer seedlings under snow cover (Matsumoto and Hsiang 2016). Therefore, snow moulds are a generic name including diverse fungi and fungal-like microbes belonging to various taxa (oomycetes, ascomycetes and basidiomycetes). Important pathogens of agricultural crops from temperate to frigid zones in the Northern Hemisphere are *Pythium* spp. (e.g. *Pythium iwayamai*) in oomycetes, *Microdochium nivale* and *Sclerotinia* spp. (e.g. *Spirorbis borealis*) in ascomycetes and *Typhula* spp. (e.g. *T. incarnata* and *Typhula ishikariensis* complex) in basidiomycetes (Hoshino and Matsumoto 2013).

The snow moulds *Pythium polare* and related species occur frequently as pathogens of moss and vascular plants species in the Arctic and Antarctic, chiefly in maritime area (Petrov 1983; Tojo and Newsham 2012; Tojo et al. 2012). Two psychrophilic snow moulds *Spirorbis borealis* and *Typhula ishikariensis* are also widely distributed not only in the cool temperate zone and frigid zone but also in Arctic regions such as Alaska and the Yukon (Lebeau and Longston 1958; McBeath 2002), Greenland (Hoshino et al. 2006), Finnmark (northern Norway: Årsvoll 1975; Matsumoto and Tronsmo 1995; Matsumoto et al. 1996), Iceland (*S. borealis* not found: Kristinsson and Guðleifsson 1976; Hoshino et al. 2004), Lapland (northern Finland and Sweden: Jamalainen 1949, 1957; Ekstrand 1955), Svalbard (Hoshino et al. 2003), Russian Arctic (Tkachenko 2013). Their distribution pattern suggests that *S. borealis* and *T. ishikariensis* are highly adapted to the Arctic environment. *S. borealis* and *T. ishikariensis* were not found from Antarctica. However, *Sclerotinia antarctica* on Antarctic hair grass *Deschampsia antarctica* was found in Antarctic peninsula. The fungus is morphologically similar to *S. borealis* (Gamundi and Spideni 1987) and needs comparisons with *S. borealis*. However, there is no *Typhula* species of snow moulds recorded from Antarctica.

Basidiomycetous hyphae were observed in dead moss leaves from both Polar regions (Wilson 1951; Tojo and Newsham 2012), and these hyphae have less taxonomical features such as conidial and other sexual stages. We collected basidiomycetous hyphae in dead moss leaves on King George Island, South Shetland Islands, maritime Antarctica, and DNA sequences of these pathogens had high homology with *Typhula* species. In this paper, we described first record of snow mould *Typhula* species and their position in Antarctica.

## Material and methods

### Collection

The specimens of hyphae from snow blight of *Polytrichum juniperinum* were collected from coastal area in Geographer cove, Fildes Peninsula, King George Island (62°02′, 58°21′W), South Shetland Islands, Antarctica. The specimens of basidiocarps of *Typhula* sp. were also collected from Handspike Corner Herb. Fields, Macquarie Island (54°30′, 158°57′E), Australia. Dates of collection are shown below. These specimens were kept in the mycological herbarium of National Museum of Nature and Science (TNS), Tokyo, Japan.

### Morphological observations

Colours of hyphae and basidiocarps were described according to the colour identification chart of the Royal Botanic Garden Edinburgh (*Flora of British Fungi*) (Royal Botanic Garden Edinburgh 1969).

For light microscopic observations of hyphae with moss shoots, dried or fresh specimens were mounted in water. For observation of hyphae in moss shoots using a transmission electron microscope (H-800, Hitachi Ltd., Tokyo, Japan), samples were prepared according to Malajczuk et al. (1977).

### Phylogenetic analyses

DNA was extracted from the specimens by the protocol of DNeasy Plant Mini (Qiagen, Hilden, Germany). The internal transcribed spacer (ITS) region of genomic rDNA was amplified with the primer pairs ITS1 (5′-TCCGTAGGTGAACCTGCGG-3′) and ITS4 (5′-TCCTCCGCTTATTGATATGC-3′), as described by Hsiang and Wu (2000). The polymerase chain reaction (PCR) product was purified using a QIAquick PCR Purification Kit (Qiagen) and sequenced on an ABI PRISM 3100 Genetic Analyzer (Applied Biosystems, Foster City, CA, USA) using the primer ITS1.

Multiple alignments of the ITS sequences were performed, and the nucleotide substitution rate was calculated. A phylogenetic tree was constructed by the neighbour-joining method (Kimura 1980; Saitou and Nei 1987) using the programme CLUSTAL W (Thompson et al. 1994) with bootstrap values based on 1000 replications (Felsenstein 1985).

## Results and discussion

### Symptoms of snow blight of Antarctic moss

**Figure 1.**
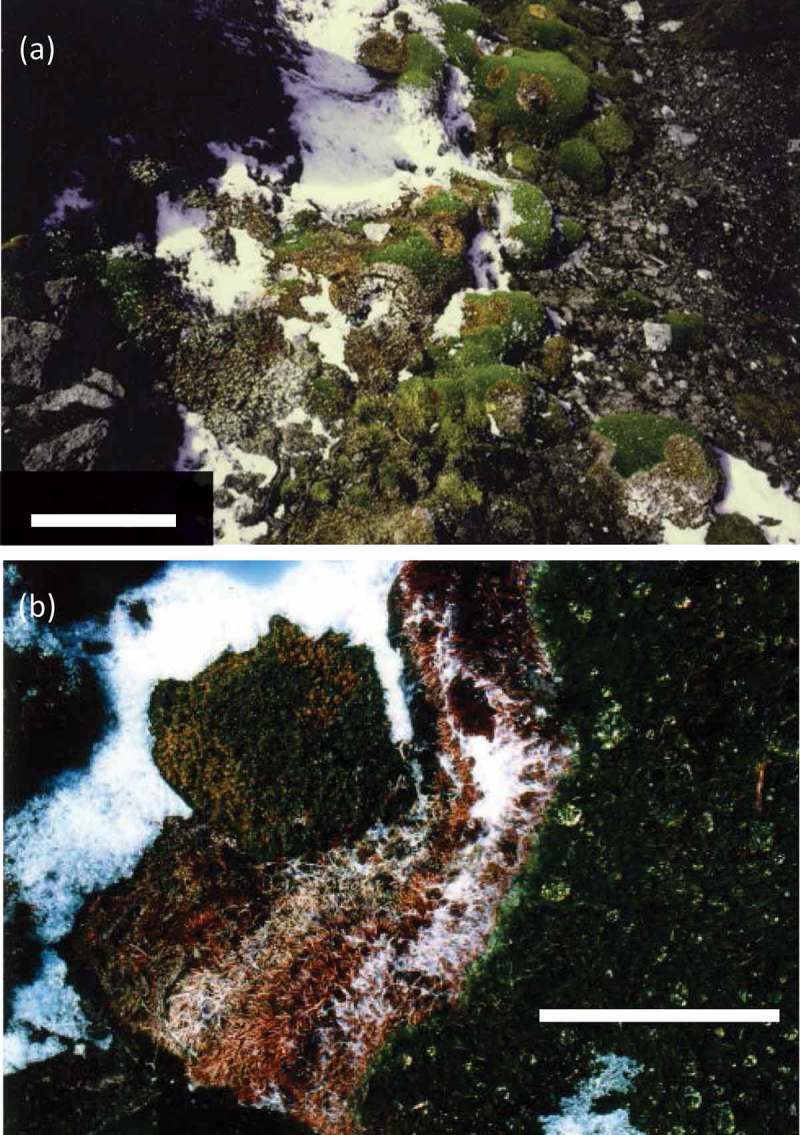
Fungal infections in *Polytrichum juniperinum* on King George Island, maritime Antarctica. Bars (a) 30 cm and (b) 5 cm.

**Figure 2.**
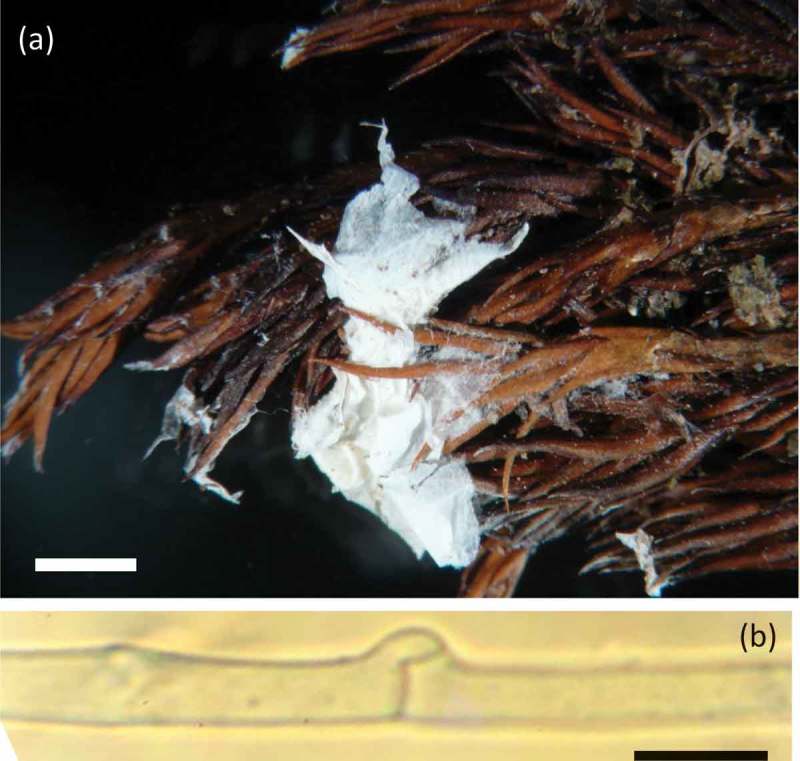
Macro and microscopic observations of fungi on moribund moss colonies (TNS-F-40295). Bars (a) 5 mm and (b) 10 µm.

The moss colonies of *P. juniperinum* were spread over wet or submerged areas near Great Wall Station (Chen et al. 1993). Many moribund moss colonies were found in *P. juniperinum* after the snow bed had melted (Figure 1). Host died in a circle of about 10–30 cm after snow melts. In our observations, many fungal infections in moss were found in moss vegetation near the seashore in the research area. However, we could not find any obvious disease in moss along the coasts of Svalbard (Hoshino et al. 1999). Fungal infections were seen in circular patterns or irregular patches, and sclerotina were not observed in these symptoms. Mycelia on moss shoots were visible to the naked eye immediately after thawing (Figures 1(b) and 2 (a)). Similar observation was reported from both Polar regions (Tojo and Newsham 2012).

**Figure 3.**
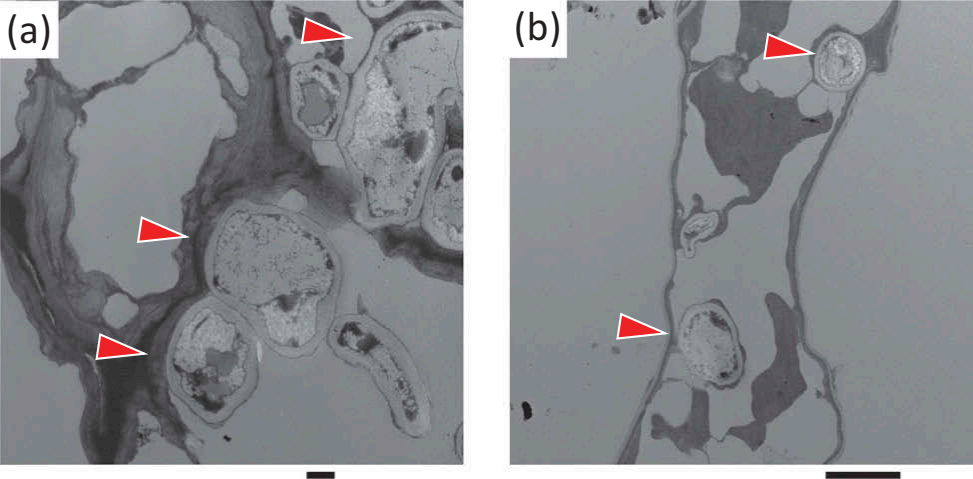
Transmission electron microscopic observation of hypha-like structures in moribund moss cells (TNS-F-40295). Arrowheads: hypha-like structures. Bars (a) 500 nm and (b) 2 µm.

Figures 2 and 3 show microscopic images of a moribund leaf of *P. juniperinum*. Clamp connected hyphae mycelia grew well on the moss leaf and were intertwisted at the growth point of the moss (Figure 2). Several numbers of hypha-like structures observed in host moss cells (Figure 3). These hypha-like structures were almost similar size of those of *T. ishikariensis* infected in bentgrass (Oshiman et al. 1995). We also obtained DNA from fungal infected moss shoots and only one kind of fungal ITS sequence from these specimens (detail was described in next section). Therefore, we regarded that one pathogenic fungus caused snow mould disease with *P. juniperinum* in Antarctica. Unfortunately, we did not success to isolate fungi in moribund moss leaves. In Arctic, Wilson (1951) studied infections of *Rhacomitrium* carpet on Jan Mayen Island and reported that the moss disease was caused by an unidentified basidomycete.

## Phylogenic analysis and morphological characteristics of *Typhula* sp. from Macquarie Island

**Figure 4.**
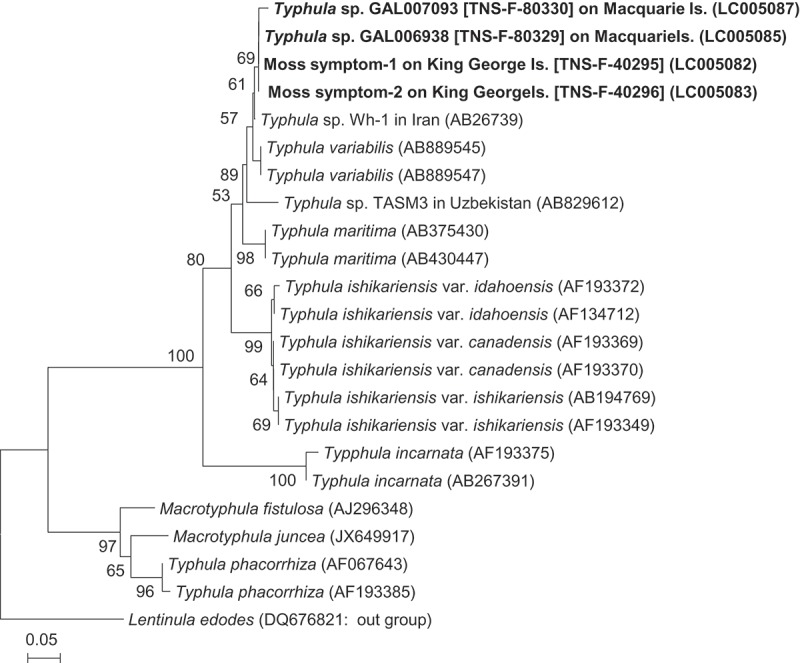
Neighbour-joining (NJ) tree based on sequences of the internal transcribed spacer 1 (ITS1)-5.8S-ITS2 region for phylogenetically related species of the genus of *Typhula*. Bootstrap percentages (from 1000 replications) greater than 50% are shown at branch points. Accession numbers are given in parentheses.

ITS sequences were obtained from two moss symptoms (LC005082 and LC005083, Table 1). Phylogenetic comparisons of our specimens with previous data on ITS sequences of *Typhula* spp. and related genera are shown in Figure 4. Fungi in moss symptoms were perfectly matched with the basidiocarp of *Typhula* sp. GAL006938 collected on Macquarie Island, Subantarctica, Australia, and was highly homology with *Typhula* sp. Wh-1 that caused snow mould disease to winter wheat in Iran (Hoshino et al. 2007). DNA sequence data also indicated that this fungus in moss cells is in the genus *Typhula*, and this is first record of *Typhula* snow blight in Antarctica.

**Table 1.** Specimens of the *Typhula* from Antarctica used in this study.

Specimen no.		Isolate origin			
Origin	International	Substrate	Locality	Collection period	Accession no.
AIST-KG1	TNS-F-40295	On dead moss (*Polytrichum*	Geographer cove, Fildes Peninsula	22 December 1997	LC005082
		*juniperinum*)	King George Is., Antarctica		
AIST-KG2	TNS-F-40296	Same substrate	Same locality	22 December 1997	LC005083
GAL006938	TNS-F-80329	On rotting leaf of *Stilbocarpa* sp.	Aerial Bay, Macquarie Is., Australia	3 March 1955	LC005085
GAL007093	TNS-F-80330	On rotting leaf of *Stilbocarpa* sp.	Aerial Bay, Macquarie Is., Australia	7 April 1955	LC005087

**Figure 5.**
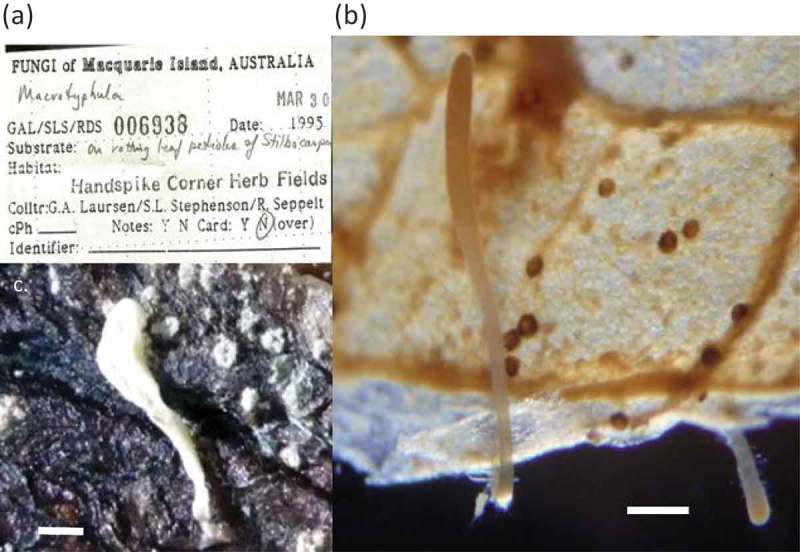
*Typhula* sp. GAL006938 (TNS-F-80329) from Macquarie Island in maritime Antarctica. (a) Specimen label. (b) Immersion specimen. (c) Dry specimen. Bars (b) and (c) 1 mm.

*Typhula* sp. GAL006938 and other specimens on Macquarie Island were regarded as saprotrophic species in maritime Antarctica. Morphological characteristics of *Typhula* sp. GAL006938 were following; Basidiocarps (Figure 5) one to per substrates, 5–8 mm high, narrowly to broadly clavate, obscurely stipitate, simple apex rounded, watery white to whitish (1A or 7 white) at dried buff to cinnamon (2B to 6F) at immersion specimen; stipe 2.5–4 mm long, 0.2–0.3 wide; head 2.5–4 mm long and 0.3–0.6 mm wide, stuffed at first then hollow, no basidia and basidiospores.

These morphological characteristics of basidiocarps of *Typhula* sp. GAL006938 and sclerotia of *Typhula* sp. Wh-1 were similar with *Typhula subvariabilis* (Berthier 1974; Berthier 1976). Several saprophytic species of *Typhula* and related genera of *Macrotyphula* and *Pistillaria* are formed basidiocarps from sclerotia and substrates. Those species are psychrotrophic and if their mycelium can survive and grow at environmental condition, they form the basidiocarps directory from substrates (Hoshino et al. unpublished results). On subantarctic islands such as King George Island (62°02′, 58°21′W) and Macquarie Island (54°30′, 158°57′E), annual ground temperature is enough low for mycelial growth of *Typhula* sp. Therefore, this fungus formed basidiocarps directory from substrates and we did not find sclerotium on these symptoms. We regarded that *Typhula* sp. caused snow mould disease on moss colonies in Antarctic Islands and this pathogen was similar with *T. subvariabils* (*T*. cf. *subvariabiris*).

## Evolution of snow moulds in Antarctica

We found fungal disease on the moss, *Polytrichum juniperium* on King George Island, Antarctica. Sclerotia were not found on diseased plants; however, molecular data suggested that *Typhula* cf. *subvariabilis* was present there. These findings suggest that three genera of representative snow moulds *Pythium, Sclerotinia* and *Typhula* could share the same niches in both Polar regions.

Other fungal species are also recognised as snow moulds in Antarctica (Tojo and Newsham 2012). Zygomycetous snow moulds are not found in the Northern Hemisphere, but *Rhizopus* sp. was pathogen on the moss *Bryum anatrcticum* at Cape Bird on Ross Island (Greenfield 1983). Unfortunately, the specimens are not available (Greenfield, personal communication). Several ascomycetes such as *Thyronectria antarctica* var. *hyperantarctica, Coleroa turfosorum, Bryosphaeria megaspora, Epibryon chorisodontii* and an unidentified Plectomycete were recorded from ring infection and macroscopic section of mosses (Hawksworth 1973; Longton 1973; Fenton 1983). Cryophilic fungi are less in the number of species than that of fungi in temperate region (Hoshino et al. 2013), and flora in Polar regions are different from other regions of cryosphere in temperate and frigid zones. Therefore, there must be new niches for cryophilic fungal phytopathogens in Polar regions, and novel cryophilic fungi are likely to have evolved as phytopathogens to adapted in polar climate and vegetation.

Pathogenicity and cold-adaptation are essential factors for fungi to become cryophilic fungal phytopathogens, and these factors co-evolved in *Typhula* (Hoshino 2005). *T. ishikariensis* complex well adapted Arctic environment. However, their basidiospores did not adapt in long-distance dispersionhave by air-borne (Cunfer and Bruehl 1973); therefore, this fungus was not found from low latitude snowy regions (Hoshino et al. 2007). Antarctica is geographically isolated from other continents. *T*. cf. *variabilis* was distributed in low latitude snowy regions such as Middle East, and their basidiospores probably succeeded to survive and adapt in the Antarctic environments. We should survey the geographical distribution of *T*. cf. *subvariabilis* around Antarctica.
